# How do case managers spend time on their functions and activities?

**DOI:** 10.1186/s12913-016-1333-6

**Published:** 2016-04-02

**Authors:** Emily (Chuanmei) You, David Dunt, Colleen Doyle

**Affiliations:** Institute for Health and Ageing; School of Nursing Midwifery & Paramedicine, Australian Catholic University (in partnership with Villa Maria Catholic Homes), Victoria, Australia; Centre for Health Policy, Melbourne School of Population and Global Health, The University of Melbourne, Victoria, Australia; School of Nursing Midwifery & Paramedicine, Australian Catholic University (in partnership with Villa Maria Catholic Homes), Victoria, Australia; National Ageing Research Institute, Victoria, Australia

**Keywords:** Activities, Case management, Community aged care, Functions, Influencing factors

## Abstract

**Background:**

Case management has been a widely accepted approach to practice in various care settings. This study aimed to explore how community aged care case managers allocated their time to case management functions, how frequently they performed specific case management activities, and what factors influenced the frequency of their activities.

**Methods:**

The study involved 154 survey participants, or 17.1 % of the target case managers in the State of Victoria, Australia. Key information collected included participants’ socio-demographic characteristics, proportions of time allocated to six core case management functions, and frequency ratings of 41 specific activities within seven case management functions. Ordinal regression analyses were performed to determine significant factors associated with participants’ frequency ratings of their activities.

**Results:**

Participants allocated the largest proportion of time to care coordination (22.0 %), and the smallest proportion of time to outcome evaluation (8.0 %). Over 70 % of the participants assigned high frequency ratings to 31 of the 41 case management activities. The remaining ten activities, including all four outcome evaluation activities, three needs assessment activities, one care planning activity, one care coordination activity, and one general functions-related activity were less commonly performed very frequently. The regression analyses indicated that some case manager and client factors were significantly associated with frequency ratings of nine of the ten activities aforementioned. The two main findings of the regression analyses were: First, emphasising achieving more case management goals was significantly associated with higher frequency of three outcome evaluation activities; second, longer work experience was significantly associated with higher frequency of one care coordination activity and one outcome evaluation activity.

**Conclusions:**

The frequent performance of most case management activities and relative absence of factors influencing their frequency suggest a uniformity of practice in community aged care case managers’ practice. What is not clear is whether the frequency of these activities (in particular less frequent performance of outcome evaluation activities) conforms to expectations.

**Electronic supplementary material:**

The online version of this article (doi:10.1186/s12913-016-1333-6) contains supplementary material, which is available to authorized users.

## Background

Case management has been a widely accepted approach to practice in community care settings, such as primary health care, community mental health, and community aged care [1]. In Australia, there have been three publicly funded community aged care case management programs/packages (Community Aged Care Package—CACP, Extended Aged Care at Home—EACH and Extended Aged Care at home Dementia—EACHD) that provide different levels of services to community-dwelling frail older Australians. Case managers manage services for a number of CACP, EACH and/or EACHD clients to ensure that they access case management support and case-managed care services (one client uses one care package) [2] (see Table 1).

**Table 1:** Case-managed community aged care programs/packages in Australia

CACP program –comparative to low residential care (since 1992)	EACH program-comparative to high residential care (since 2002)	EACHD program-comparative to high residential care (since 2006)
CACP clients	EACH clients	EACHD clients
1) Frail older people aged 70 and over (50 and over if indigenous) 2) Preferring & being able to live in the community 3) Assessed as eligible for low residential care 4) Complex care needs resulting from physical, social & psychological conditions 5) Needing comprehensive care management & ongoing monitoring and review of their changing care needs.	1) Similar characteristics of CACP clients 2) High care needs & assessed as eligible for high residential care.	1) Similar characteristics of EACH clients 2) Behavioural & psychological symptoms of dementia (BPSD).
CACP services	EACH services	EACHD services
1) Personal care (such as bathing & dressing) 2) Domestic care (such as housework, shopping, meal preparation & gardening) 3) Social support 4) Transport to appointments.	1) CACP services 2) Carer support 3) Allied health 4) Clinical nursing care 5) Certain mobility equipment 6) Continence consumables.	1) EACH services 2) Special care services (such as dementia care) for addressing clients’ BPSD problems.
Case management support, functions and activities		
Typical case management support: 1) Needs assessment 2) Care planning 3) Identifying services that will best meet clients’ needs 4) Arranging additional services through brokerage (brokering/purchasing services from external agencies or persons) or advocacy 5) Monitoring ongoing needs & service delivery 6) Staff management related to clients (excluding rostering) 7) Liaising with family members & other professionals such as general practitioners 8) Administering packages, including leave, collection of fees & suspension of services and case closure if necessary. Typical case management functions/activities: 1) Developing, monitoring & formally reviewing care plans 2) Coordinating & negotiating services provided by internal & external aged care organisations 3) Providing assistance for clients and carers, e.g. understanding and managing situations, behaviours and relationships; providing emotional support; assisting individuals to access and use general community services/facilities (advocacy); providing one-to-one training or advice 4) Communicating with & providing information (such as available services) to clients’ carers/family members.		

Note: 1. All three programs are funded by the Commonwealth Government of Australia. 2. Data sources: [2, 36–38]

The literature has consistently agreed that primary case management functions include needs assessment, care planning, implementation, coordination, and monitoring and review [3, 4]. It is difficult, however, to reach consensus on specific case management activities within these functions across care settings and health systems of different countries. Some U.S. studies have investigated different numbers and types of case management activities in various community care settings, or in one particular care setting, such as community mental health and community nursing [5–9]. In addition, a British study has investigated eight case management elements adopted by practice nurses working in the community [10].

Some studies have indicated that outcome evaluation is also an important case management function [11–13], but there are debates on whether it is an independent function or it overlaps or is part of the *monitoring and review* function in individual case managers’ practice [13]. While some empirical studies (examining frequency ratings of different case management activities) have reported that in their practice case managers perform outcome evaluation activities less frequently than the other case management activities [5, 8, 9], some empirical [13] and commentary studies [12, 14] have revealed that case managers do not perform outcome evaluation adequately because they lack time, knowledge of goals/outcomes and goal setting, capacities, and organisational support.

There has been a paucity of studies based in community aged care that focus on how case managers allocate time to the primary case management functions, or how frequently they undertake those functions and specific case management activities. Some studies have reported that case managers spend considerable time on care coordination as well as more time on initial assessment compared with monitoring and review [14–16]. However, these studies examined different case management function domains (such as direct care, indirect case management, and program management), and/or were based in other care settings but not specifically in community aged care.

In light of the case management approach being employed in various care settings and case management in community aged care having different features in terms of specific target populations, interventions, and approaches to delivering services, how community aged care case managers distribute their time across different functions and activities is of considerable interest [17]. In addition, given the complexity and busy nature of the case management profession, it is of paramount importance for case managers to make the best use of their time in those functions and activities they value. This may improve their work efficiency and the outcome of care [14, 18].

There has been even less research exploring factors that affect case management functions and activities. Some research and review studies have reported that client factors (such as some socio-demographic characteristics and health condition), case manager factors (such as professional backgrounds and length of employment), organisational factors (such as financial restrictions and other organisational policies), and system factors (such as available system resources) influence case managers’ performance of specific functions and activities [3, 19–22], or the overall case management practice [13]. Such great variability in the factors that influence case managers’ practice indicates the necessity to examine significant factors associated with case managers’ functions and/or activities by empirical research.

This study aimed to investigate Australian community aged care case managers’ functions and activities, and significant factors associated with their activities. The research questions included: How do case managers allocate time to case management functions? How frequently do case managers undertake specific case management activities? What are the significant factors associated with case managers’ frequency of performance of their activities? Is outcome evaluation performed frequently by case managers?

We hypothesised that individual characteristics (such as age, gender, caseloads, professional backgrounds etc.), clients’ characteristics (such as percentages of clients living with dementia, living alone etc.) and organisational factors (including size and attributes) would be significantly associated with case managers’ frequency of performance of their activities.

## Methods

This study was part of a larger project (case management practice, goals and outcomes in community aged care: perspectives of case managers in Australia) implemented between November 2010 and April 2014 [13]. The quantitative research presented here involved a survey of case managers working for community aged care organisations in Victoria Australia.

Conducted between September 2012 and February 2013, the survey collected information on participants’ characteristics, proportions of their time allocated to case management functions, and their frequency ratings (never, seldom, occasionally, often, and very often) of 41 activities within seven case management functions, including needs assessment (ten activities), care planning (eight activities), care plan implementation (three activities), care coordination (six activities), monitoring and review (five activities), outcome evaluation (four activities), and general functions (five activities).

### Study setting and population

Eligible participants for the survey were case managers who managed a number of CACP, EACH, and/or EACHD clients in Victoria. Because there was no official data about the case management workforce at the national and state levels, we calculated the study population in Victoria by using the total number of community aged care clients (13,468 in June 2011) and the ratio of clients to case managers (15:1) [23]. Therefore, the estimated study population at this period of time was 898 (13468/15). However, we may have overestimated this number because we found that case managers on average managed 21 clients (see Table 2).

**Table 2:** Descriptive information on case managers, and their clients and organisations

Variables	n (%)	Mean (range)	SD
Case manager information			
1. Age			
18–40	42 (27.3)		
41–60	98 (63.6)		
Over 60	14 (9.1)		
2. Female gender	134 (87.0)		
3. Case managers (versus lead case managers)	130 (84.8)		
4. Australian born (versus born overseas)	110 (71.4)		
5. Professional backgrounds			
Nursing	45 (29.2)		
Social work	38 (24.7)		
Allied health	47 (30.5)		
Others	24 (15.6)		
6. Qualifications			
Certificate/diploma	59 (38.3)		
Bachelor	79 (51.3)		
Master	16 (10.4)		
7. Practice locations			
Rural place	39 (25.3)		
Suburb	73 (47.5)		
City	21 (13.6)		
Multiple regions	21 (13.6)		
8. Full-time (versus casual/part-time) workers	94 (61.4)		
9. Level of authority over budget management			
No to partial	99 (65.6)		
Complete	52 (34.4)		
10. Level of involvement in organisational decision-making			
No to medium	119 (81.0)		
Strong	28 (19.0)		
11. Years working as a case manager		6.4 (0.2–30)	5.4
12. Caseload: number of community aged care (CACP, EACH and EACHD) and other community care clients		21 (3–45)	7.4
13. Caseload types			
Only one type	37 (24.8)		
More than one type	112 (75.2)		
14. Number of community aged care clients		18 (3–40)	8.8
15. Emphasis on achieving the number of goals		4.9 (1–7)	1.4
Organisational information			
1. Organisational size (measured by the number of community aged care packages provided)		414 (10–1298)	442
2. Private not-for-profit (versus government-owned) organisations	116 (75.3)		
Client information			
1. Born in Australia (%)		57.2 % (0–100 %)	30.6 %
2. Living with dementia (%)		21.6 % (0–92.6 %)	16.3 %
3. Living alone (%)		47.8 % (0–100 %)	22.6 %
4. Having carers (%)		50.2 % (0–100 %)	28.5 %
5. Having high care needs (%)		34.3 % (0–100 %)	24.4 %
6. Level of involvement in case management processes			
No to mild	29 (19.6)		
Moderate	53 (35.8)		
Strong	66 (44.6)		

Note: 1. The sample size was not 154 for some variables due to missing data

### Survey recruitment and the study sample

To invite all 898 case managers to participate, we contacted all 110 community aged care organisations in Victoria via email, phone, mail and fax. We also made presentations in some organisations to invite participation. Ultimately, 154 case managers (17.1 %) from 55 organisations (50 %) participated.

### Development of the survey questionnaire

The development of the survey questionnaire was informed by a comprehensive literature review (journal articles, government and professional bodies’ guidelines, and available job descriptions of case managers in some organisations [2, 4, 5, 24–26]), discussions among the authors, consultations with the Melbourne School of Population and Global Health Human Ethics Advisory Group, and a pilot survey (prior to the formal survey) with five experienced case managers.

### Measures of this study

- **Dependent variables**
  Dependent variables were frequency ratings of 41 case management activities.
- **Independent variables**
  Based on the literature review [3, 8, 19–21] and available information, independent variables included in ordinal logistic regression analyses were:
  - **Organisational factors**: attributes and size.
  - **Case manager factors**: age, gender, job titles, birthplaces, practice locations, years working as a case manager, professional backgrounds, qualifications, employment status, level of authority over budget management, level of involvement in organisational decision-making, caseload managed (number of community aged care and other community care clients), number of community aged care clients managed, caseload types (one vs. more than one type), and emphasis on achieving the number of case management goals.
  - **Client factors**: born in Australia, living with dementia, living alone, having carers, having high care needs, and level of involvement in case management processes.

### Data analysis

In the larger project, the following formula was used to calculate the adequate sample size: *n* = N × Z^2^ × r × (1-r)/((N-1) × E^2^ + Z^2^ × r × (1-r)) [27]. “n” is the estimated minimum sample size; “N” is the estimated population size 898 (see above); “r” is the response rate (50 %) of our interest; “Z” is the critical value (1.96) for the confidence level (95 %); “E” is the margin of error. While researchers normally use a margin of error of 5 % (hence *n* = 269), we used 8 % (hence *n* = 129). This might reduce the reliability of the survey results; however, 8 % falls between the acceptable levels of margin of error (4–8 %) [28]. In addition, it was more practical for us to recruit a smaller number of participants.

Descriptive statistics, F tests, and ordinal logistic regression analyses were conducted using PASW 19.0. Since case management practice is multifaceted and attracts professionals from a wide range of professional backgrounds such as nursing and social work [29], we hypothesised that professional backgrounds might be associated with case managers’ performance of their activities. Therefore, we analysed participants’ frequency ratings of 41 activities by profession.

To determine significant factors (significance level set at 0.05) associated with frequency ratings of each activity, we conducted two steps of regression analyses. First, we performed univariate regression analyses to identify potential significant factors (significance level set at 0.1) associated with frequency ratings of each activity. Next, we performed multivariate regression analyses (only examining those potential significant factors) to identify the final significant factors associated with frequency ratings of each activity. Eventually, we identified significant factors for nine activities. We reported 95 % confidence intervals and proportional odds ratios for the significant results.

The project was approved by the Melbourne School of Population and Global Health Human Ethics Advisory Group, The University of Melbourne (approval number 1237778).

## Results

On average, organisations involved in the survey provided 414 community aged care packages (range: 10–1298; SD = 442). Most (75.3 %) were private not-for-profit organisations. Case managers were generally middle-aged females from a range of professional backgrounds, and the majority of them (75.2 %) managed mixed types of clients (see Table 2).

### Proportions of time allocated to different case management functions

Participants on average allocated 22 % of time to care coordination and 16.5 % to monitoring and review per month (see Table 3). They allocated about 13 % of time to care plan implementation, care planning, and needs assessment respectively. By contrast, they allocated only 8 % of time to outcome evaluation and other program-related functions respectively.

**Table 3:** Proportions of time allocated to case management functions per month

	Mean (range)	SD	F	*p*
Needs assessment			**1.256**	**0.292**
Nursing case managers (*n* = 42)	14.0 % (3.0 %–30.0 %)	7.1 %		
Social worker case managers (*n* = 36)	12.3 % (0–20.0 %)	6.6 %		
Allied health case managers (*n* = 42)	11.9 % (3.5 %–25.0 %)	5.3 %		
Case managers with other professional backgrounds (*n* = 24)	11.3 % (5.0 %–20.0 %)	4.9 %		
Total (*N* = 144)	12.5 % (0–30.0 %)	6.2 %		
Care planning			**3.171**	**0.026**
Nursing case managers (*n* = 42)	15.3 % (2.0 %–40.0 %)	8.3 %		
Social worker case managers (*n* = 36)	12.1 % (1.0 %–20.0 %)	5.4 %		
Allied health case managers (*n* = 42)	13.4 % (5.0 %–30.0 %)	6.2 %		
Case managers with other professional backgrounds (*n* = 24)	10.6 % (5.0 %–20.0 %)	4.0 %		
Total (*N* = 144)	13.2 % (1.0 %–40.0 %)	6.5 %		
Care plan implementation			**0.095**	**0.963**
Nursing case managers (*n* = 42)	13.8 % (1.0 %–30.0 %)	6.4 %		
Social worker case managers (*n* = 36)	13.8 % (5.0 %–30.0 %)	6.7 %		
Allied health case managers (*n* = 42)	14.2 % (2.5 %–40.0 %)	7.6 %		
Case managers with other professional backgrounds (*n* = 24)	13.3 % (3.0 %–30.0 %)	6.2 %		
Total (*N* = 144)	13.8 % (1.0 %–40.0 %)	6.8 %		
Care coordination			**0.497**	**0.685**
Nursing case managers (*n* = 42)	20.4 % (1.0 %–50.0 %)	13.0 %		
Social worker case managers (*n* = 36)	22.8 % (4.0 %–90.0 %)	17.2 %		
Allied health case managers (*n* = 42)	21.3 % (0–50.0 %)	13.6 %		
Case managers with other professional backgrounds (*n* = 24)	24.6 % (5.0 %–50.0 %)	12.8 %		
Total (*N* = 144)	22.0 % (0–90.0 %)	14.2 %		
Monitoring and review			**0.398**	**0.755**
Nursing case managers (*n* = 42)	15.4 % (5.0 %–30.0 %)	6.8 %		
Social worker case managers (*n* = 36)	16.4 % (0.5 %–50.0 %)	9.7 %		
Allied health case managers (*n* = 42)	17.0 % (4.0 %–40.0 %)	9.6 %		
Case managers with other professional backgrounds (*n* = 24)	17.7 % (10.0 %–50.0 %)	9.3 %		
Total (*N* = 144)	16.5 % (0.5 %–50.0 %)	8.8 %		
Outcome evaluation			**1.490**	**0.220**
Nursing case managers (*n* = 42)	8.8 % (0–20.0 %)	4.0 %		
Social worker case managers (*n* = 36)	7.0 % (0–25.0 %)	5.3 %		
Allied health case managers (*n* = 42)	7.4 % (0–20.0 %)	4.9 %		
Case managers with other professional backgrounds (*n* = 24)	9.0 % (0–20.0 %)	4.7 %		
Total (*N* = 144)	8.0 % (0–25.0 %)	4.8 %		
Other functions (program-related)			**0.117**	**0.950**
Nursing case managers (*n* = 42)	8.2 % (0–50.0 %)	10.1 %		
Social worker case managers (*n* = 36)	9.1 % (0–60.0 %)	14.2 %		
Allied health case managers (*n* = 42)	7.6 % (0–50.0 %)	10.3 %		
Case managers with other professional backgrounds (*n* = 24)	8.2 % (0–55.0 %)	12.4 %		
Total (*N* = 144)	8.3 % (0–60.0 %)	11.6 %		
Other functions (not program-related)			**0.185**	**0.907**
Nursing case managers (*n* = 42)	5.0 % (0–77.0 %)	12.4 %		
Social worker case managers (*n* = 36)	3.9 % (0–15.0 %)	4.6 %		
Allied health case managers (*n* = 42)	5.3 % (0–60.0 %)	10.4 %		
Case managers with other professional backgrounds (*n* = 24)	4.4 % (0–15.0 %)	4.7 %		
Total (*N* = 144)	4.7 % (0–77.0 %)	9.2 %		

Note:1. The sample size was 144 due to missing data. 2. Significance level was set at 0.05

Table 3 also shows that professional backgrounds were not significantly associated with case managers allocating their time to most functions except care planning. For the latter, nursing case managers on average allocated significantly a higher proportion of their time (*F* = 3.17; *p* = 0.026).

### Frequency ratings assigned to specific activities within different functions

Over 70 % of participants assigned high frequency ratings (often or very often) to 31 of the 41 activities (see Additional file 1).

Less than 70 % of the participants assigned high frequency ratings to the remaining ten activities which are presented in Table 4. These included three needs assessment activities (F1, F2, and F3), one care planning activity (F11), one care coordination activity (F22), all four outcome evaluation activities (F33, F34, F35 and F36), and one general functions-related activity (F37). The three activities that were less commonly conducted very frequently were: “F1-identify potential clients for case management programs (45.0 %),” “F33-develop implementation plans for evaluating the effects (41.5 %),” and “F37-generate client summary reports and present them to key stakeholders (50.7 %)”.

**Table 4:** Frequency ratings of some case management activities by profession

Case management activities	Never	Seldom	Occasionally	Often	Very often	High frequency rating (often or very often)
	n (%)	n (%)	n (%)	n (%)	n (%)	n (%)
**F1**-identify potential clients requiring case management services						
Nursing case managers (*n* = 45)	3 (6.7)	5 (11.1)	15 (33.3)	11 (24.4)	11 (24.4)	22 (48.8)
Social worker case managers (*n* = 37)	2 (5.4)	8 (21.6)	11 (29.7)	11 (29.7)	5 (13.5)	16 (43.2)
Allied health case managers (*n* = 46)	5 (10.9)	9 (19.6)	13 (28.3)	9 (19.6)	10 (21.7)	19 (41.3)
Case managers with other professional backgrounds (*n* = 23)	5 (21.7)	3 (13.0)	4 (17.4)	6 (26.1)	5 (21.7)	11 (47.8)
**Total (** ***N*** **= 151)**	**15 (9.9)**	**25 (16.6)**	**43 (28.5)**	**37 (24.5)**	**31 (20.5)**	**68 (45.0)**
**F2**-organise secondary specialist assessment for clients if necessary						
Nursing case managers (*n* = 45)		2 (4.4)	13 (28.9)	21 (46.7)	9 (20.0)	30 (66.7)
Social worker case managers (*n* = 37)		1 (2.7)	12 (32.4)	18 (48.6)	6 (16.2)	24 (64.8)
Allied health case managers (*n* = 45)	1 (2.2)		18 (40.0)	20 (44.4)	6 (13.3)	26 (57.7)
Case managers with other professional backgrounds (*n* = 23)			7 (30.4)	12 (52.2)	4 (17.4)	16 (69.6)
**Total (** ***N*** **= 150)**	**1 (0.7)**	**3 (2.0)**	**50 (33.3)**	**71 (47.3)**	**25 (16.7)**	**96 (64.0)**
**F3**-assess clients’ readiness and willingness for case management services						
Nursing case managers (*n* = 45)	1 (2.2)		8 (17.8)	26 (57.8)	10 (22.2)	36 (80.0)
Social worker case managers (*n* = 37)		8 (21.6)	11 (29.7)	9 (24.3)	9 (24.3)	18 (48.6)
Allied health case managers (*n* = 46)	2 (4.3)	4 (8.7)	9 (19.6)	17 (37.0)	14 (30.4)	31 (67.4)
Case managers with other professional backgrounds (*n* = 23)		1 (4.3)	4 (17.4)	14 (60.9)	4 (17.4)	18 (78.3)
**Total (** ***N*** **= 151)**	**3 (2.0)**	**13 (8.6)**	**32 (21.2)**	**66 (43.7)**	**37 (24.5)**	**103 (68.2)**
**F7**-assess clients’ relationships with key stakeholders (e.g. care providers, family members, carers, etc.)						
Nursing case managers (*n* = 45)	1 (2.2)		2 (4.4)	16 (35.6)	26 (57.8)	42 (93.4)
Social worker case managers (*n* = 37)			1 (2.7)	15 (40.5)	21 (56.8)	36 (97.3)
Allied health case managers (*n* = 46)	3 (6.5)		7 (15.2)	17 (37.0)	19 (41.3)	36 (78.3)
Case managers with other professional backgrounds (*n* = 23)	1 (4.3)		5 (21.7)	11 (47.8)	6 (26.1)	17 (73.9)
**Total (** ***N*** **= 151)**	**5 (3.3)**		**15 (9.9)**	**59 (39.1)**	**72 (47.7)**	**131 (86.8)**
**F11**-conduct research on the availability of resources (particularly financial and care resources) and then develop care plans based on the research findings						
Nursing case managers (*n* = 45)	5 (11.1)	8 (17.8)	6 (13.3)	16 (35.6)	10 (22.2)	26 (57.8)
Social worker case managers (*n* = 36)	2 (5.6)	8 (22.2)	8 (22.2)	7 (19.4)	11 (30.6)	18 (50.0)
Allied health case managers (*n* = 45)	1 (2.2)	5 (11.1)	12 (26.7)	19 (42.2)	8 (17.8)	27 (60.0)
Case managers with other professional backgrounds (*n* = 23)	1 (4.3)	2 (8.7)	3 (13.0)	13 (56.5)	4 (17.4)	17 (73.9)
**Total (** ***N*** **= 149)**	**9 (6.0)**	**23 (15.4)**	**29 (19.5)**	**55 (36.9)**	**33 (22.1)**	**88 (59.0)**
**F13**-assess barriers that may influence achieving expected goals and then determine corresponding strategies						
Nursing case managers (*n* = 45)		1 (2.2)	1 (2.2)	20 (44.4)	23 (51.1)	43 (95.5)
Social worker case managers (*n* = 36)		1 (2.8)	5 (13.9)	12 (33.3)	18 (50.0)	30 (83.3)
Allied health case managers (*n* = 45)	2 (4.4)	1 (2.2)	8 (17.8)	19 (42.2)	15 (33.3)	34 (75.5)
Case managers with other professional backgrounds (*n* = 23)		1 (4.3)		12 (52.2)	10 (43.5)	22 (95.7)
**Total (** ***N*** **= 149)**	**2 (1.3)**	**4 (2.7)**	**14 (9.4)**	**63 (42.3)**	**66 (44.3)**	**129 (86.6)**
**F22**-provide or facilitate to provide health prevention/education services to improve clients and carers’ wellness						
Nursing case managers (*n* = 45)			17 (37.8)	21 (46.7)	7 (15.6)	28 (62.3)
Social worker case managers (*n* = 35)	2 (5.7)	4 (11.4)	11 (31.4)	11 (31.4)	7 (20.0)	18 (51.4)
Allied health case managers (*n* = 44)		2 (4.5)	22 (50.0)	12 (27.3)	8 (18.2)	20 (45.5)
Case managers with other professional backgrounds (*n* = 22)		2 (9.1)	10 (45.5)	8 (36.4)	2 (9.1)	10 (45.5)
**Total (** ***N*** **= 146)**	**2 (1.4)**	**8 (5.5)**	**60 (41.1)**	**52 (35.6)**	**24 (16.4)**	**76 (52.0)**
**F29**-monitor clients’ progress in terms of achieving expected outcomes at specific time frames as defined by care plans						
Nursing case managers (*n* = 45)			3 (6.7)	21 (46.7)	21 (46.7)	42 (93.4)
Social worker case managers (*n* = 36)	2 (5.6)	1 (2.8)	7 (19.4)	14 (38.9)	12 (33.3)	26 (72.2)
Allied health case managers (*n* = 44)			5 (11.4)	28 (63.6)	11 (25.0)	39 (88.6)
Case managers with other professional backgrounds (*n* = 22)			2 (9.1)	13 (59.1)	7 (31.8)	20 (90.9)
**Total (** ***N*** **= 147)**	**2 (1.4)**	**1 (0.7)**	**17 (11.6)**	**76 (51.7)**	**51 (34.7)**	**127 (86.4)**
**F33**-develop implementation plans for evaluating the effects of case management plans systematically and periodically						
Nursing case managers (*n* = 45)	6 (13.3)	5 (11.1)	8 (17.8)	16 (35.6)	10 (22.2)	26 (57.8)
Social worker case managers (*n* = 36)	7 (19.4)	7 (19.4)	10 (27.8)	9 (25.0)	3 (8.3)	12 (33.3)
Allied health case managers (*n* = 44)	9 (20.5)	10 (22.7)	13 (29.5)	10 (22.7)	2 (4.5)	12 (27.2)
Case managers with other professional backgrounds (*n* = 22)	2 (9.1)	4 (18.2)	5 (22.7)	8 (36.4)	3 (13.6)	11 (50.0)
**Total (** ***N*** **= 147)**	**24 (16.3)**	**26 (17.7)**	**36 (24.5)**	**43 (29.3)**	**18 (12.2)**	**61 (41.5)**
**F34**-evaluate multicultural issues and other factors that influence achieving identified goals and expected outcomes						
Nursing case managers (*n* = 45)	1 (2.2)	7 (15.6)	13 (28.9)	13 (28.9)	11 (24.4)	24 (53.3)
Social worker case managers (*n* = 36)	2 (5.6)	1 (2.8)	10 (27.8)	16 (44.4)	7 (19.4)	23 (63.8)
Allied health case managers (*n* = 44)	2 (4.5)	6 (13.6)	9 (20.5)	17 (38.6)	10 (22.7)	27 (61.3)
Case managers with other professional backgrounds (*n* = 22)	1 (4.5)	1 (4.5)	6 (27.3)	10 (45.5)	4 (18.2)	14 (63.7)
**Total (** ***N*** **= 147)**	**6 (4.1)**	**15 (10.2)**	**38 (25.9)**	**56 (38.1)**	**32 (21.8)**	**88 (59.9)**
**F35**-evaluate the effects related to identified goals and expected outcomes (e.g. client outcomes, carer outcomes, cost-effectiveness, cost-benefits, etc.) at specified timeframes as defined by care plans						
Nursing case managers (*n* = 45)	1 (2.2)	2 (4.4)	8 (17.8)	16 (35.6)	18 (40.0)	34 (75.6)
Social worker case managers (*n* = 36)	3 (8.3)	4 (11.1)	8 (22.2)	12 (33.3)	9 (25.0)	21 (58.3)
Allied health case managers (*n* = 44)	3 (6.8)	3 (6.8)	10 (22.7)	20 (45.5)	8 (18.2)	28 (63.7)
Case managers with other professional backgrounds (*n* = 22)		1 (4.5)	5 (22.7)	13 (59.1)	3 (13.6)	16 (72.7)
**Total (** ***N*** **= 147)**	**7 (4.8)**	**10 (6.8)**	**31 (21.1)**	**61 (41.5)**	**38 (25.9)**	**99 (67.4)**
**F36**-evaluate the feasibility, timeliness, availability, quality and appropriateness of services identified in care plans						
Nursing case managers (*n* = 45)	2 (4.4)	3 (6.7)	5 (11.1)	21 (46.7)	14 (31.1)	35 (77.8)
Social worker case managers (*n* = 36)	3 (8.3)	5 (13.9)	5 (13.9)	9 (25.0)	14 (38.9)	23 (63.9)
Allied health case managers (*n* = 44)	3 (6.8)	3 (6.8)	11 (25.0)	15 (34.1)	12 (27.3)	27 (61.4)
Case managers with other professional backgrounds (*n* = 22)		3 (13.6)	3 (13.6)	11 (50.0)	5 (22.7)	16 (72.7)
**Total (** ***N*** **= 147)**	**8 (5.4)**	**14 (9.5)**	**24 (16.3)**	**56 (38.1)**	**45 (30.6)**	**101 (68.7)**
**F37**-generate client summary reports and present them to key stakeholders (e.g. care professionals, care providers, payers, etc.)						
Nursing case managers (*n* = 44)	4 (9.1)	7 (15.9)	9 (20.5)	17 (38.6)	7 (15.9)	24 (54.5)
Social worker case managers (*n* = 36)	1 (2.8)	9 (25.0)	7 (19.4)	15 (41.7)	4 (11.1)	19 (52.8)
Allied health case managers (*n* = 45)	5 (11.1)	5 (11.1)	15 (33.3)	9 (20.0)	11 (24.4)	20 (44.4)
Case managers with other professional backgrounds (*n* = 23)	2 (8.7)	3 (13.0)	6 (26.1)	8 (34.8)	4 (17.4)	12 (52.2)
**Total (** ***N*** **= 148)**	**12 (8.1)**	**24 (16.2)**	**37 (25.0)**	**49 (33.1)**	**26 (17.6)**	**75 (50.7)**
**F38**-document the processes of all the core functions as described above and communicate related information to key stakeholders (e.g. clients and carers, care professionals, care providers, payers, etc.)						
Nursing case managers (*n* = 44)	4 (9.1)	1 (2.3)	4 (9.1)	14 (31.8)	21 (47.7)	35 (79.5)
Social worker case managers (*n* = 36)	1 (2.8)		3 (8.3)	11 (30.6)	21 (58.3)	32 (88.9)
Allied health case managers (*n* = 45)	1 (2.2)		2 (4.4)	6 (13.3)	36 (80.0)	42 (93.3)
Case managers with other professional backgrounds (*n* = 23)			1 (4.3)	8 (34.8)	14 (60.9)	22 (95.7)
**Total (** ***N*** **= 148)**	**6 (4.1)**	**1 (0.7)**	**10 (6.8)**	**39 (26.4)**	**92 (62.2)**	**131 (88.6)**

Note: 1. The sample size was not 154 due to missing data. 2. This table includes activities (F1, F2, F3, F11, F22, F33, F34, F35, F36, and F37) that less than 70 % of participants assigned high frequency ratings and activities (F3, F7, F11, F13, F22, F29, F33, F34, F36 and F38) that participants from different professional backgrounds assigned obviously different frequency ratings

Through presenting the data by profession, we identified four key findings: First, higher proportions of nursing case managers assigned high frequency ratings to 27 activities (see Additional file 1), in particular “F3-assess client readiness and willingness”, “F22-provide or facilitate to provide health prevention/education services”, “F33-develop implementation plans for evaluating the effects”, and “F36-evaluate service feasibility, timeliness, etc”. However, much lower proportions of nursing case managers assigned high frequency ratings to “F34-evaluate multicultural issues and other influencing factors for goal attainment” and “F38-document the processes and communicate information to key stakeholders”. (See Table 4).

Second, lower proportions of allied health case managers assigned high frequency ratings to 18 activities (see Additional file 1), in particular “F3-assess client readiness and willingness”, “F13-assess barriers hindering goal attainment,” and “F33-develop implementation plans for evaluating the effects”. (See Table 4).

Third, close to average proportions of social work case managers assigned high frequency ratings to most activities (see Additional file 1) except “F3-assess client readiness and willingness (far below average)”, “F29-monitor clients’ health progress (far below average)”, and “F7-assess client relationships with key stakeholders (far above average) (see Table 4).

Fourth, higher proportions of case managers with other professional backgrounds assigned high frequency ratings to 10 activities (see Additional file 1), in particular “F11-conduct research on resources and then develop care plans”. (See Table 4).

### Significant factors associated with frequency ratings of nine activities

The two steps of regression analyses determined potential significant factors (see Additional file 1) and final significant factors (see Table 5) associated with frequency ratings of nine activities. The significant results are summarised below:

**Table 5:** Significant factors associated with frequency ratings of nine case management activities

	OR	P	95 % CI
**F1**-identify potential clients requiring case management services			
Job titles		0.042	
Lead case managers	2.66	0.042	1.04–6.82
Case managers (reference group)	1.00		
Gender		0.027	
Male	0.36	0.027	0.14–0.90
Female (reference group)	1.00		
**F2**-organise secondary specialist assessment for clients if necessary			
Percentage of clients with high care needs	1.01	0.049	1.00–1.03
Involvement in organisational decision-making		0.016	
Strong	2.94	0.016	1.22–7.03
None to medium (reference group)	1.00		
**F11**-conduct research on the availability of resources (particularly financial and care resources) and then develop care plans based on the research findings			
Percentage of clients with carers	1.02	0.010	1.00–1.03
Age		0.069	
Over 60	4.35	0.036	1.11–16.95
41–60	0.70	0.350	0.34–1.48
18–40 (reference group)	1.00		
**F22**-provide or facilitate to provide health prevention/education services to improve clients and carers’ wellness			
Years working as a case manager	1.09	0.009	1.02–1.16
Birthplaces		0.004	
Overseas	3.25	0.004	1.46–7.24
Australia (reference group)	1.00		
Practice locations		0.102	
Multiple locations	0.24	0.014	0.08–0.75
Suburb	0.58	0.216	0.24–1.38
City	0.70	0.534	0.22–2.18
Rural area (reference group)	1.00		
**F33**-develop implementation plans for evaluating the effects of case management plans systematically and periodically			
Number of case management goals	1.28	0.022	1.04–1.58
**F34**-evaluate multicultural issues and other factors that influence achieving identified goals and expected outcomes			
Number of case management goals	1.28	0.033	1.02–1.60
Years working as a case manager	1.07	0.027	1.01–1.14
**F35**-evaluate the effects related to identified goals and expected outcomes (e.g. client outcomes, carer outcomes, cost-effectiveness, cost-benefits, etc.) at specified timeframes as defined by care plans			
Number of case management goals	1.27	0.033	1.02–1.57
Educational level		0.087	
Master	0.29	0.026	0.10–0.86
Bachelor	0.51	0.069	0.24–1.05
Diploma/certificates (reference group)	1.00		
Authority over budget management		0.016	
Complete	2.48	0.016	1.19–5.21
None or partial (reference group)	1.00		
**F36**-evaluate the feasibility, timeliness, availability, quality and appropriateness of services identified in care plans			
Employment status		0.017	
Full-time	2.16	0.017	1.15–4.10
Casual or part-time (reference group)	1.00		
**F37**-generate client summary reports and present them to key stakeholders (e.g. care professionals, care providers, payers, etc.)			
Age		0.002	
Over 60	0.15	0.001	0.05–0.45
41-60	0.56	0.092	0.28–0.91
18-40 (reference group)	1.00		

Note: 1. In both univariate and multivariate analyses, to ensure more than 10 in the contingency table of outcome by covariate [39], some data were merged for some outcome measures. For example, “never”, “seldom”, and “occasionally” were merged for frequency ratings of F1, F22, F34, F35 and F36. “Never” and “seldom” were merged for frequency ratings of F2. 2. *OR* Odds Ratio, *CI* Confidence Interval. 3. Significance level was set at 0.05

### Two needs assessment activities

Lead case managers (OR = 2.66) and female participants (OR = 2.78) were more likely to assign high frequency ratings to “F1-identify potential clients for case management programs.” Participants having greater percentage of clients with high care needs (OR = 1.01) and participants with stronger involvement in organisational decision-making (OR = 2.94) were more likely to assign high frequency ratings to “F2-organise secondary specialist assessment for clients”.

### One care planning activity

Participants having higher percentage of clients with carers (OR = 1.02) and participants aged over 60 (OR = 4.35) were more likely to assign high frequency ratings to “F11-conduct research on resources and then develop care plans”.

### One care coordination activity

Participants working longer as case managers (OR = 1.09) and participants born overseas (OR = 3.25) were more likely to assign high frequency ratings to “F22-provide or facilitate to provide health prevention/education”.

### One general functions-related activity

Participants aged over 60 (OR = 0.15) were less likely to assign high frequency ratings to “F37-generate client summary reports and present them to stakeholders”.

### Four outcome evaluation activities

Participants focusing on achieving more case management goals (OR = 1.28) were more likely to give high frequency ratings to “F33-develop implementation plans for evaluating the effects”. Participants focusing on achieving more case management goals (OR = 1.28) and participants working longer as case managers (OR = 1.07) were more likely to assign high frequency ratings to “F34-evaluate multicultural issues and other influencing factors for goal attainment”. Participants focusing on achieving more case management goals (OR = 1.27) and participants having complete authority to manage budgets (OR = 2.48) were more likely to assign high frequency ratings to “F35-evaluate the effects related to goals and outcomes”. Participants working full time (OR = 2.16) were more likely to assign high frequency ratings to “F36-evaluate service feasibility, timeliness etc”.

## Discussion

Unsurprisingly, we found that case managers allocated the largest proportion of time to care coordination. In practice a large part of case managers’ daily activities is to frequently communicate and negotiate with care professionals, clients and families, and significant others, so as to coordinate right services at the right time for clients [16].

Contrary to the literature [10, 14, 15], we found that case managers allocated a higher proportion of time to monitoring and review (16.5 %) than needs assessment (12.5 %). In addition, the majority of case managers (over 70 %) assigned high frequency ratings to all five monitoring and review activities but not all needs assessment activities. Further research is needed to determine reasons for different findings identified by our study.

We found that case managers allocated the lowest proportion of time to outcome evaluation, and assigned lower frequency ratings to all four outcome evaluation activities compared with most other case management activities. These findings are partially consistent with those U.S. studies discussed in the Background section [5, 8, 9], though bear in mind that they focused on various care settings and examined different numbers and types of outcome evaluation activities. It is worth conducting new research to explore the reasons for these findings.

We found that a very low proportion of case managers assigned high frequency ratings to “identify potential clients requiring case management services”. This finding might reflect the fact in the Australian context that Aged Care Assessment Teams rather than case managers assess a person’s eligibility for a case management program [2]. We also found that a very low proportion of case managers assigned high frequency ratings to “generate client summary reports and present them to key stakeholders”. We were unable to refer this finding to the literature. New research might explore this issue further.

Interestingly, the majority of case managers assigned high frequency ratings to 31 of the 41 case management activities, implying that there is considerable consistency in case managers’ frequency performance of most activities in their practice examined by this study.

It is not surprising to find that emphasising achieving more case management goals was significantly associated with higher frequency ratings of three outcome evaluation activities. Researchers have argued that case managers’ activities should be outcome/goal-directed (e.g. achieving various client and organisational goals) [13, 30], and undertaking outcome evaluation activities will facilitate case managers to achieve desired goals and outcomes [13, 31].

We found that longer work experience was significantly associated with case managers’ higher frequency ratings of two activities: “provide or facilitate to provide health prevention and education services to improve clients and carers’ wellness” and “evaluate multicultural issues and other factors that influence achieving identified goals and expected outcomes”. These findings partially support previous studies, revealing that experienced case managers are more likely to adopt dementia care practice (case screening, using assessment tools and undertaking interventions) [22] and are more culturally responsive (regarded as an advanced capacity) in practice [32]. However, it is difficult to interpret those findings that some case manager factors (such as age and practice locations) and some client factors (such as having high care needs and having carers) were only significantly associated with frequency ratings of one activity, respectively.

Our study showed that higher proportions of case managers with a nursing background (compared with social work, allied health and other professional backgrounds) assigned high frequency ratings to most activities (27 in 41). Since nursing case managers have more medical knowledge and skills, and are more medically task-oriented and goal-oriented, they might be more capable of undertaking some activities (such as medical assessments and outcome evaluation) or more willing to perform some activities (such as health prevention and education) that they can make the best use of their knowledge and skills [13]. Conversely, our study found that lower proportions of allied health case managers assigned high frequency ratings to many activities (18 of 41). These indirectly support previous findings that organisations expect allied health case managers to apply their expertise in undertaking some non-typical case management activities (such as functioning assessments and psychotherapies) to contribute to their team and improvements in clients’ health [13, 15].

### Limitations

The low participant response rate (17.1 %) may impact on participant representativeness of this study. Besides those strategies we used to invite participation (calls, emails, mails, fax and site visits), it is worth exploring more strategies to increase the response rate in future studies.

Because we contacted organisations to invite their case managers to participate in our study, some individuals might have participated out of organisational influence. Thus the quality of the information they provided might have been affected.

Quantitative studies emphasise reliability and validity. Constrained by resources and time, we only focused on improving the validity. These included determining the sample frame and calculating the sample size prior to the survey, endeavouring to increase the response rate, designing the questionnaire based on existing evidence and expert consultations, and piloting the questionnaire.

Lastly, we did not collect individual clients’ information or more organisational information, limiting our ability to identify other client and organisational factors that might be significantly associated with case managers’ practice.

### Implications

Without existing evidence, this study sets a precedent in exploring how community aged care case managers allocated their time (measured by proportions) to different case management functions and specific case management activities (measured by frequency ratings). These findings were generated from a sample of front-line case managers’ perceptions and therefore can provide practical guidance for case managers’ practice.

The significant correlation of longer work experience and the possible correlation of professional backgrounds with case managers’ frequency performance of their activities suggest that having a professional background may be related to reporting more frequent interventions. Further research is needed to determine the meaning of reporting more frequent interventions, and the benefits to clients of having a professional case manager.

The survey questionnaire and findings can assist organisations and governments to develop practice guidelines and standards for case managers working in community aged care sectors [8, 33, 34]. Moreover, the questionnaire has the potential to be converted into an assessment tool. This can be further used to measure functions and activities of case managers working in commuity aged care and similar care settings [8, 34]. The survey findings can also assist organisations to design case managers’ job descriptions. The latter will facilitate case managers to set professional boundaries in the delivery of case management interventions and services, and benefit the organisations in candidate selection and staff members’ orientation and ongoing development [35].

## Conclusions

Both the frequent performance of most case management activities and relative absence of associated significant factors are interesting new findings adding to the literature on case management practice. These findings suggest a uniformity of practice in community aged care case managers. Whether outcome evaluation activities by nature should be performed less frequently or are underperformed by individual case managers could be the topic of further research in the area.

## Additional file

Additional file 1: Table S1. Potential significant factors associated with frequency ratings of nine case management activities. **Table S2.** Frequency ratings of all 41 case management activities by profession. (DOCX 59 kb)

## Abbreviations

CACP: Community Aged Care Package
EACH: Extended Aged Care at Home
EACHD: Extended Aged Care at Home Dementia
