# Supplementary material for: How do case managers spend time on their functions and activities?
Source: BMC Health Serv Res. 2016 Apr 2;16:112. doi: 10.1186/s12913-016-1333-6 (PMC4818942; doi:10.1186/s12913-016-1333-6)
Supplement: Additional file 1: Table S1. — Potential significant factors associated with frequency ratings of nine case management activities. Table S2. Frequency ratings of all 41 case management activities by profession. (DOCX 59 kb) [file 12913_2016_1333_MOESM1_ESM.docx]

**Additional file 1**

Table S1. Potential significant factors associated with frequency ratings of nine case management activities

| Case management activities | Potential significant factors |
| --- | --- |
| **Needs assessment** | |
| F1. Identify potential clients requiring case management services | Organisational attributes (p=0.04), job titles (p=0.009), gender (p=0.051), birthplaces (p=0.09), and involvement in organisational decision-making (p=0.035) |
| F2. Organise secondary specialist assessment for clients if necessary | Years working as a case manager (p=0.069), educational level (p=0.098), involvement in organisational decision-making (p=0.019), and percentage of clients having high care needs (p=0.054) |
| Care planning | |
| F11. Conduct research on the availability of resources (particularly financial and care resources) and then develop care plans based on the research findings | Age (p=0.024), involvement in organisational decision-making (p=0.078), percentage of clients living alone (p=0.047), percentage of clients having carers (p=0.004), and client involvement in case management processes (p=0.074) |
| Care coordination | |
| F22. Provide or facilitate to provide health prevention/education services to improve clients and carers’ wellness | Birthplaces (p=0.038), practice locations (p=0.01), years working as a case manager (p=0.007), caseloads (p=0.083), client involvement in case management processes (p=0.038), and number of case management goals (p=0.098) |
| Outcome evaluation | |
| F33. Develop implementation plans for evaluating the effects of case management plans systematically and periodically | Organisational attributes (p=0.046), age (p=0.016), birthplaces (p=0.065), practice locations (p=0.078), number of community aged care packages (p=0.008), caseload types (p=0.083), and number of case management goals (p=0.035) |
| F34. Evaluate multicultural issues and other factors that influence achieving identified goals and expected outcomes | Organisational attributes (p=0.014), birthplaces (p=0.053), years working as a case manager (p=0.018), employment status (p=0.01), percentage of clients born in Australia (p=0.076), percentage of clients with carers (p=0.073), and number of case management goals (p=0.009) |
| F35. Evaluate the effects related to identified goals and expected outcomes (e.g. client outcomes, carer outcomes, cost-effectiveness, cost-benefits, etc.) at specified timeframes as defined by care plans | Practice locations (p=0.021), educational level (p=0.098), authority to manage budgets (p=0.001), involvement in organisational decision-making (p=0.004), client involvement in case management processes (p=0.003), and number of case management goals (p=0.034) |
| F36. Evaluate the feasibility, timeliness, availability, quality and appropriateness of services identified in care plans | Employment status (p=0.004), authority to manage budgets (p=0.042), and number of case management goals (p=0.081) |
| General functions-related activities | |
| F37. Generate client summary reports and present them to key stakeholders (e.g. care professionals, care providers, payers, etc.) | Organisational attributes (p=0.096) and age (p=0.001) |

Note: 1. In univariate ordinal regression analyses, significance level was set at 0.1. In other words, a variable with p value less than 0.1 in the regression analysis result was identified as a potential significant factor. 2. In both univariate and multivariate analyses, to ensure more than 10 in the contingency table of outcome by covariate [[37](#_ENREF_37)], some data were merged for some outcome measures. For example, “never”, “seldom”, and “occasionally” were merged for frequency ratings of F1, F22, F34, F35 and F36. “Never” and “seldom” were merged for frequency ratings of F2.

**Table S2. Frequency ratings of all 41 case management activities by profession**

| **Activities** | | **Never**  **n(%)** | **Seldom**  **n(%)** | **Occasionally**  **n(%)** | **Often**  **n(%)** | **Very often**  **n(%)** | **High frequency rating (often or very often)**  **n(%)** |
| --- | --- | --- | --- | --- | --- | --- | --- |
| **Needs assessment** | | | | | | | |
| **F1. Identify potential clients requiring case management services** | | | | | | | |
| Nursing case managers (n=45) | | 3 (6.7) | 5 (11.1) | 15 (33.3) | 11 (24.4) | 11 (24.4) | 22 (48.8) |
| Social worker case managers (n=37) | | 2 (5.4) | 8 (21.6) | 11 (29.7) | 11 (29.7) | 5 (13.5) | 16 (43.2) |
| Allied health case managers (n=46) | | 5 (10.9) | 9 (19.6) | 13 (28.3) | 9 (19.6) | 10 (21.7) | 19 (41.3) |
| Case managers with other professional backgrounds (n=23) | | 5 (21.7) | 3 (13.0) | 4 (17.4) | 6 (26.1) | 5 (21.7) | 11 (47.8) |
| **Total (N=151)** | | **15 (9.9)** | **25 (16.6)** | **43 (28.5)** | **37 (24.5)** | **31 (20.5)** | **68 (45.0)** |
| **F2. Organise secondary specialist assessment for clients if necessary** | | | | | | | |
| Nursing case managers (n=45) | |  | 2 (4.4) | 13 (28.9) | 21 (46.7) | 9 (20.0) | 30 (66.7) |
| Social worker case managers (n=37) | |  | 1 (2.7) | 12 (32.4) | 18 (48.6) | 6 (16.2) | 24 (64.8) |
| Allied health case managers (n=45) | | 1 (2.2) |  | 18 (40.0) | 20 (44.4) | 6 (13.3) | 26 (57.7) |
| Case managers with other professional backgrounds (n=23) | |  |  | 7 (30.4) | 12 (52.2) | 4 (17.4) | 16 (69.6) |
| **Total (N=150)** | | **1 (0.7)** | **3 (2.0)** | **50 (33.3)** | **71 (47.3)** | **25 (16.7)** | **96 (64.0)** |
| **F3. Assess clients’ readiness and willingness for case management services** | | | | | | | |
| Nursing case managers (n=45) | | 1 (2.2) |  | 8 (17.8) | 26 (57.8) | 10 (22.2) | 36 (80.0) |
| Social worker case managers (n=37) | |  | 8 (21.6) | 11 (29.7) | 9 (24.3) | 9 (24.3) | 18 (48.6) |
| Allied health case managers (n=46) | | 2 (4.3) | 4 (8.7) | 9 (19.6) | 17 (37.0) | 14 (30.4) | 31 (67.4) |
| Case managers with other professional backgrounds (n=23) | |  | 1 (4.3) | 4 (17.4) | 14 (60.9) | 4 (17.4) | 18 (78.3) |
| **Total (N=151)** | | **3 (2.0)** | **13 (8.6)** | **32 (21.2)** | **66 (43.7)** | **37 (24.5)** | **103 (68.2)** |
| **F4.** **Assess clients’ ability to participate in case management processes** | | | | | | | |
| Nursing case managers (n=45) | | 1 (2.2) |  | 6 (13.3) | 24 (53.3) | 14 (31.1) | 38 (84.4) |
| Social worker case managers (n=37) | |  |  | 11 (29.7) | 14 (37.8) | 12 (32.4) | 26 (70.2) |
| Allied health case managers (n=46) | | 3 (6.5) | 2 (4.3) | 7 (15.2) | 17 (37.0) | 17 (37.0) | 34 (74.0) |
| Case managers with other professional backgrounds (n=23) | |  | 1 (4.3) | 5 (21.7) | 10 (43.5) | 7 (30.4) | 17 (73.9) |
| **Total (N=151)** | | **4 (2.6)** | **3 (2.0)** | **29 (19.2)** | **65 (43.0)** | **50 (33.1)** | **115 (76.1)** |
| **F5.** **Assess carers’ needs and conditions where applicable** | | | | | | | |
| Nursing case managers (n=45) | |  | 1 (2.2) | 4 (8.9) | 18 (40.0) | 22 (48.9) | 40 (88.9) |
| Social worker case managers (n=37) | |  |  | 8 (21.6) | 15 (40.5) | 14 (37.8) | 29 (78.3) |
| Allied health case managers (n=45) | |  | 2 (4.4) | 6 (13.3) | 26 (57.8) | 11 (24.4) | 37 (82.2) |
| Case managers with other professional backgrounds (n=23) | |  | 1 (4.3) | 5 (21.7) | 9 (39.1) | 8 (34.8) | 17 (73.9) |
| **Total (N=150)** | |  | **4 (2.7)** | **23 (15.3)** | **68 (45.3)** | **55 (36.7)** | **123 (82.0)** |
| **F6.** **Identify clients’ additional/alternate types or levels of care needs** | | | | | | | |
| Nursing case managers (n=45) | | 1 (2.2) |  | 4 (8.9) | 22 (48.9) | 18 (40.0) | 40 (88.9) |
| Social worker case managers (n=37) | |  | 1 (2.7) | 3 (8.1) | 18 (48.6) | 15 (40.5) | 33 (89.1) |
| Allied health case managers (n=46) | | 2 (4.3) | 1 (2.2) | 7 (15.2) | 20 (43.5) | 16 (34.8) | 36 (78.3) |
| Case managers with other professional backgrounds (n=23) | |  |  | 3 (13.0) | 13 (56.5) | 7 (30.4) | 20 (86.9) |
| **Total (N=151)** | | **3 (2.0)** | **2 (1.3)** | **17 (11.3)** | **73 (48.3)** | **56 (37.1)** | **129 (85.4)** |
| **F7.** **Assess clients’ relationships with key stakeholders (e.g. care providers, family members, carers, etc.)** | | | | | | | |
| Nursing case managers (n=45) | | 1 (2.2) |  | 2 (4.4) | 16 (35.6) | 26 (57.8) | 42 (93.4) |
| Social worker case managers (n=37) | |  |  | 1 (2.7) | 15 (40.5) | 21 (56.8) | 36 (97.3) |
| Allied health case managers (n=46) | | 3 (6.5) |  | 7 (15.2) | 17 (37.0) | 19 (41.3) | 36 (78.3) |
| Case managers with other professional backgrounds (n=23) | | 1 (4.3) |  | 5 (21.7) | 11 (47.8) | 6 (26.1) | 17 (73.9) |
| **Total (N=151)** | | **5 (3.3)** | **0** | **15 (9.9)** | **59 (39.1)** | **72 (47.7)** | **131 (86.8)** |
| **F8.** **Assess clients’ health conditions, functional status, psychological status, psychosocial status, and risk factors** | | | | | | | |
| Nursing case managers (n=45) | | 1 (2.2) |  |  | 18 (40.0) | 26 (57.8) | 44 (97.8) |
| Social worker case managers (n=37) | |  |  | 3 (8.1) | 9 (24.3) | 25 (67.6) | 34 (91.9) |
| Allied health case managers (n=46) | | 2 (4.3) | 1 (2.2) | 5 (10.9) | 20 (43.5) | 18 (39.1) | 38 (82.6) |
| Case managers with other professional backgrounds (n=23) | |  |  | 1 (4.3) | 13 (56.5) | 9 (39.1) | 22 (95.6) |
| **Total (N=151)** | | **3 (2.0)** | **1 (0.7)** | **9 (6.0)** | **60 (39.7)** | **78 (51.7)** | **138 (91.4)** |
| **F9.** **Use established case management process and standards to perform the assessment** | | | | | | | |
| Nursing case managers (n=45) | |  | 1 (2.2) |  | 13 (28.9) | 31 (68.9) | 44 (97.8) |
| Social worker case managers (n=37) | |  |  | 4 (10.8) | 11 (29.7) | 22 (59.5) | 33 (89.2) |
| Allied health case managers (n=45) | | 1 (2.2) |  | 5 (11.1) | 15 (33.3) | 24 (53.3) | 39 (86.6) |
| Case managers with other professional backgrounds (n=23) | |  |  |  | 14 (60.9) | 9 (39.1) | 23 (100.0) |
| **Total (N=150)** | | **1 (0.7)** | **1 (0.7)** | **9 (6.0)** | **53 (35.3)** | **86 (57.3)** | **139 (92.6)** |
| **F10.** **Assess clients’ social support system, financial resources, environmental conditions (e.g. living situation), multicultural issues, and other factors that may impact on their conditions** | | | | | | | |
| Nursing case managers (n=45) | |  | 1 (2.2) | 1 (2.2) | 17 (37.8) | 26 (57.8) | 43 (95.6) |
| Social worker case managers (n=37) | |  |  | 3 (8.1) | 13 (35.1) | 21 (56.8) | 34 (91.9) |
| Allied health case managers (n=46) | |  |  | 3 (6.5) | 18 (39.1) | 25 (54.3) | 43 (93.4) |
| Case managers with other professional backgrounds (n=23) | |  |  |  | 13 (56.5) | 10 (43.5) | 23 (100.0) |
| **Total (N=151)** | |  | **1 (0.7)** | **7 (4.6)** | **61 (40.4)** | **82 (54.3)** | **143 (94.7)** |
| **Care planning** | | | | | | | |
| **F11. Conduct research on the availability of resources (particularly financial and care resources) and then develop care plans based on the research findings** | | | | | | | |
| Nursing case managers (n=45) | | 5 (11.1) | 8 (17.8) | 6 (13.3) | 16 (35.6) | 10 (22.2) | 26 (57.8) |
| Social worker case managers (n=36) | | 2 (5.6) | 8 (22.2) | 8 (22.2) | 7 (19.4) | 11 (30.6) | 18 (50.0) |
| Allied health case managers (n=45) | | 1 (2.2) | 5 (11.1) | 12 (26.7) | 19 (42.2) | 8 (17.8) | 27 (60.0) |
| Case managers with other professional backgrounds (n=23) | | 1 (4.3) | 2 (8.7) | 3 (13.0) | 13 (56.5) | 4 (17.4) | 17 (73.9) |
| **Total (N=149)** | | **9 (6.0)** | **23 (15.4)** | **29 (19.5)** | **55 (36.9)** | **33 (22.1)** | **88 (59.0)** |
| **F12.** **Use evidence-based practice guidelines in developing care plans** | | | | | | | |
| Nursing case managers (n=45) | | 2 (4.4) | 1 (2.2) | 4 (8.9) | 17 (37.8) | 21 (46.7) | 38 (84.5) |
| Social worker case managers (n=36) | | 1 (2.8) | 3 (8.3) | 5 (13.9) | 16 (44.4) | 11 (30.6) | 27 (75.0) |
| Allied health case managers (n=45) | | 2 (4.4) | 2 (4.4) | 6 (13.3) | 19 (42.2) | 16 (35.6) | 35 (77.8) |
| Case managers with other professional backgrounds (n=23) | | 1 (4.3) | 1 (4.3) | 2 (8.7) | 13 (56.5) | 6 (26.1) | 19 (82.6) |
| **Total (N=149)** | | **6 (4.0)** | **7 (4.7)** | **17 (11.4)** | **65 (43.6)** | **54 (36.2)** | **119 (79.8)** |
| **F13.** **Assess barriers that may influence achieving expected goals and then determine corresponding strategies** | | | | | | | |
| Nursing case managers (n=45) | |  | 1 (2.2) | 1 (2.2) | 20 (44.4) | 23 (51.1) | 43 (95.5) |
| Social worker case managers (n=36) | |  | 1 (2.8) | 5 (13.9) | 12 (33.3) | 18 (50.0) | 30 (83.3) |
| Allied health case managers (n=45) | | 2 (4.4) | 1 (2.2) | 8 (17.8) | 19 (42.2) | 15 (33.3) | 34 (75.5) |
| Case managers with other professional backgrounds (n=23) | |  | 1 (4.3) |  | 12 (52.2) | 10 (43.5) | 22 (95.7) |
| **Total (N=149)** | | **2 (1.3)** | **4 (2.7)** | **14 (9.4)** | **63 (42.3)** | **66 (44.3)** | **129 (86.6)** |
| **F14.** **Engage clients and carers in developing care plans and setting short-term and long-term goals** | | | | | | | |
| Nursing case managers (n=45) | | 1 (2.2) |  | 2 (4.4) | 14 (31.1) | 28 (62.2) | 42 (93.3) |
| Social worker case managers (n=36) | |  |  | 2 (5.6) | 8 (22.2) | 26 (72.2) | 36 (94.4) |
| Allied health case managers (n=45) | |  | 1 (2.2) | 2 (4.4) | 12 (26.7) | 30 (66.7) | 42 (93.4) |
| Case managers with other professional backgrounds (n=23) | |  |  | 1 (4.3) | 10 (43.5) | 12 (52.2) | 22 (95.7) |
| **Total (N=149)** | | **1 (0.7)** | **1 (0.7)** | **7 (4.7)** | **44 (29.5)** | **96 (64.4)** | **140 (93.9)** |
| **F15.** **Keep clients and carers updated with care plans and provide them information to enable them to make informed decisions** | | | | | | | |
| Nursing case managers (n=45) | | 1 (2.2) |  |  | 11 (24.4) | 33 (73.3) | 44 (97.7) |
| Social worker case managers (n=36) | | 1 (2.8) |  |  | 16 (44.4) | 19 (52.8) | 35 (97.2) |
| Allied health case managers (n=45) | | 1 (2.2) |  | 4 (8.9) | 11 (24.4) | 29 (64.4) | 40 (88.8) |
| Case managers with other professional backgrounds (n=22) | |  |  | 1 (4.5) | 9 (40.9) | 12 (54.5) | 21 (95.4) |
| **Total (N=148)** | | **3 (2.0)** |  | **5 (3.4)** | **47 (31.8)** | **93 (62.8)** | **140 (94.6)** |
| **F16.** **Verify appropriateness of the level of services as defined in care plans and liaise with appropriate professionals and care providers** | | | | | | | |
| Nursing case managers (n=45) | |  |  |  | 21 (46.7) | 24 (53.3) | 45 (100.0) |
| Social worker case managers (n=36) | | 1 (2.8) |  |  | 15 (41.7) | 20 (55.6) | 35 (97.3) |
| Allied health case managers (n=45) | | 1 (2.2) | 1 (2.2) | 3 (6.7) | 17 (37.8) | 23 (51.1) | 40 (88.9) |
| Case managers with other professional backgrounds (n=22) | |  |  | 2 (9.1) | 7 (31.8) | 13 (59.1) | 20 (90.9) |
| **Total (N=148)** | | **2 (1.4)** | **1 (0.7)** | **5 (3.4)** | **60 (40.5)** | **80 (54.1)** | **140 (94.6)** |
| **F17.** **Respond to clients’ own goals and work with clients and carers to discuss care plans to address identified needs** | | | | | | | |
| Nursing case managers (n=45) | |  |  | 1 (2.2) | 12 (26.7) | 32 (71.1) | 44 (97.8) |
| Social worker case managers (n=36) | |  |  | 1 (2.8) | 10 (27.8) | 25 (69.4) | 35 (97.2) |
| Allied health case managers (n=45) | | 1 (2.2) |  | 1 (2.2) | 14 (31.1) | 29 (64.4) | 43 (95.5) |
| Case managers with other professional backgrounds (n=23) | |  |  |  | 8 (34.8) | 15 (65.2) | 23 (100.0) |
| **Total (N=149)** | | **1 (0.7)** |  | **3 (2.0)** | **44 (29.5)** | **101 (67.8)** | **145 (97.3)** |
| **Care plan implementation** | | | | | | | |
| **F18. Plan for clients' transition along the continuum of care** | | | | | | | |
| Nursing case managers (n=45) | | 1 (2.2) |  | 7 (15.6) | 16 (35.6) | 21 (46.7) | 37 (82.3) |
| Social worker case managers (n=36) | | 1 (2.8) |  | 10 (27.8) | 11 (30.6) | 14 (38.9) | 25 (69.5) |
| Allied health case managers (n=44) | | 1 (2.3) | 2 (4.5) | 9 (20.5) | 24 (54.5) | 8 (18.2) | 32 (72.7) |
| Case managers with other professional backgrounds (n=22) | |  | 1 (4.5) | 5 (22.7) | 10 (45.5) | 6 (27.3) | 16 (72.8) |
| **Total (N=147)** | | **3 (2.0)** | **3 (2.0)** | **31 (21.1)** | **61 (41.5)** | **49 (33.3)** | **110 (74.8)** |
| **F19. Ascertain the priority of each care plan and implement them gradually** | | | | | | | |
| Nursing case managers (n=45) | | 1 (2.2) | 1 (2.2) | 7 (15.6) | 22 (48.9) | 14 (31.1) | 36 (80.0) |
| Social worker case managers (n=36) | | 3 (8.3) |  | 6 (16.7) | 14 (38.9) | 13 (36.1) | 27 (75.0) |
| Allied health case managers (n=44) | | 3 (6.8) | 4 (9.1) | 4 (9.1) | 19 (43.2) | 14 (31.8) | 33 (75.0) |
| Case managers with other professional backgrounds (n=22) | | 1 (4.5) |  | 2 (9.1) | 12 (54.5) | 7 (31.8) | 19 (86.3) |
| **Total (N=147)** | | **8 (5.4)** | **5 (3.4)** | **19 (12.9)** | **67 (45.6)** | **48 (32.7)** | **115 (78.3)** |
| **F20. Analyse the possible effects of each care plan (such as client benefits and cost) and implement cost-effective case management strategies** | | | | | | | |
| Nursing case managers (n=45) | |  |  | 4 (8.9) | 16 (35.6) | 25 (55.6) | 41 (91.2) |
| Social worker case managers (n=36) | |  | 2 (5.6) | 5 (13.9) | 11 (30.6) | 18 (50.0) | 29 (80.6) |
| Allied health case managers (n=44) | | 2 (4.5) | 3 (6.8) | 5 (11.4) | 17 (38.6) | 17 (38.6) | 34 (77.2) |
| Case managers with other professional backgrounds (n=22) | |  | 1 (4.5) | 1 (4.5) | 10 (45.5) | 10 (45.5) | 20 (91.0) |
| **Total (N=147)** | | **2 (1.4)** | **6 (4.1)** | **15 (10.2)** | **54 (36.7)** | **70 (47.6)** | **124 (84.3)** |
| **F21. Analyse the feasibility of implementing each care plan** | | | | | | | |
| Nursing case managers (n=45) | |  | 1 (2.2) | 1 (2.2) | 22 (48.9) | 21 (46.7) | 43 (95.6) |
| Social worker case managers (n=36) | |  | 1 (2.8) | 6 (16.7) | 14 (38.9) | 15 (41.7) | 29 (80.6) |
| Allied health case managers (n=44) | | 1 (2.3) | 3 (6.8) | 4 (9.1) | 17 (38.6) | 19 (43.2) | 36 (81.8) |
| Case managers with other professional backgrounds (n=22) | |  | 1 (4.5) | 2 (9.1) | 11 (50.0) | 8 (36.4) | 19 (86.4) |
| **Total (N=147)** | | **1 (0.7)** | **6 (4.1)** | **13 (8.8)** | **64 (43.5)** | **63 (42.9)** | **127 (86.4)** |
| **Care coordination** | | | | | | | |
| **F22. Provide or facilitate to provide health prevention/education services to improve clients and carers’ wellness** | | | | | | | |
| Nursing case managers (n=45) | |  |  | 17 (37.8) | 21 (46.7) | 7 (15.6) | 28 (62.3) |
| Social worker case managers (n=35) | 2 (5.7) | 4 (11.4) | 11 (31.4) | 11 (31.4) | 7 (20.0) | 18 (51.4) |  |
| Allied health case managers (n=44) |  | 2 (4.5) | 22 (50.0) | 12 (27.3) | 8 (18.2) | 20 (45.5) |  |
| Case managers with other professional backgrounds (n=22) |  | 2 (9.1) | 10 (45.5) | 8 (36.4) | 2 (9.1) | 10 (45.5) |  |
| **Total (N=146)** | **2 (1.4)** | **8 (5.5)** | **60 (41.1)** | **52 (35.6)** | **24 (16.4)** | **76 (52.0)** |  |
| **F23. Organise resources and exert efforts to coordinate and integrate various care delivery systems (e.g. aged care, health care, etc.)** | | | | | | |  |
| Nursing case managers (n=45) |  |  | 7 (15.6) | 19 (42.2) | 19 (42.2) | 38 (84.4) |  |
| Social worker case managers (n=35) |  | 1 (2.9) | 7 (20.0) | 10 (28.6) | 17 (48.6) | 27 (77.2) |  |
| Allied health case managers (n=44) |  | 4 (9.1) | 8 (18.2) | 22 (50.0) | 10 (22.7) | 32 (72.7) |  |
| Case managers with other professional backgrounds (n=22) |  | 1 (4.5) | 7 (31.8) | 10 (45.5) | 4 (18.2) | 14 (63.7) |  |
| **Total (N=146)** |  | **6 (4.1)** | **29 (19.9)** | **61 (41.8)** | **50 (34.2)** | **111 (76.0)** |  |
| **F24. Advocate for clients and negotiate extra benefits for them if necessary** | | | | | | |  |
| Nursing case managers (n=45) | 1 (2.2) | 1 (2.2) | 4 (8.9) | 17 (37.8) | 22 (48.9) | 39 (86.7) |  |
| Social worker case managers (n=35) |  |  | 7 (20.0) | 14 (40.0) | 14 (40.0) | 28 (80.0) |  |
| Allied health case managers (n=44) | 1 (2.3) | 1 (2.3) | 9 (20.5) | 22 (50.0) | 11 (25.0) | 33 (75.0) |  |
| Case managers with other professional backgrounds (n=22) |  | 1 (4.5) | 2 (9.1) | 14 (63.6) | 5 (22.7) | 19 (86.3) |  |
| **Total (N=146)** | **2 (1.4)** | **3 (2.1)** | **22 (15.1)** | **67 (45.9)** | **52 (35.6)** | **119 (81.5)** |  |
| **F25. Establish good working relationships with referral resources** | | | | | | |  |
| Nursing case managers (n=45) | 1 (2.2) |  | 2 (4.4) | 14 (31.1) | 28 (62.2) | 42 (93.3) |  |
| Social worker case managers (n=36) | 1 (2.8) |  | 4 (11.1) | 12 (33.3) | 19 (52.8) | 31 (86.1) |  |
| Allied health case managers (n=44) |  |  | 4 (9.1) | 21 (47.7) | 19 (43.2) | 40 (90.9) |  |
| Case managers with other professional backgrounds (n=22) |  |  | 3 (13.6) | 11 (50.0) | 8 (36.4) | 19 (86.4) |  |
| **Total (N=147)** | **2 (1.4)** | **0** | **13 (8.8)** | **58 (39.5)** | **74 (50.3)** | **132 (89.8)** |  |
| **F26. Identify care resources and initiate referrals as defined by care plans for corresponding clients** | | | | | | |  |
| Nursing case managers (n=45) |  | 1 (2.2) | 2 (4.4) | 20 (44.4) | 22 (48.9) | 42 (93.3) |  |
| Social worker case managers (n=35) |  |  | 5 (14.3) | 12 (34.3) | 18 (51.4) | 30 (85.7) |  |
| Allied health case managers (n=44) | 1 (2.3) |  | 3 (6.8) | 24 (54.5) | 16 (36.4) | 40 (90.9) |  |
| Case managers with other professional backgrounds (n=22) |  |  | 2 (9.1) | 7 (31.8) | 13 (59.1) | 20 (90.9) |  |
| **Total (N=146)** | **1 (0.7)** | **1 (0.7)** | **12 (8.2)** | **63 (43.2)** | **69 (47.3)** | **132 (90.5)** |  |
| **F27. Maintain a close rapport with clients and carers to have full understanding of their circumstances and complex needs** | | | | | | |  |
| Nursing case managers (n=45) |  | 1 (2.2) |  | 5 (11.1) | 39 (86.7) | 44 (97.8) |  |
| Social worker case managers (n=36) |  |  | 1 (2.8) | 8 (22.2) | 27 (75.0) | 35 (97.2) |  |
| Allied health case managers (n=44) | 1 (2.3) |  | 1 (2.3) | 12 (27.3) | 30 (68.2) | 42 (95.5) |  |
| Case managers with other professional backgrounds (n=22) |  |  |  | 10 (45.5) | 12 (54.5) | 22 (100.0) |  |
| **Total (N=147)** | **1 (0.7)** | **1 (0.7)** | **2 (1.4)** | **35 (23.8)** | **108 (73.5)** | **143 (97.3)** |  |
| **Monitoring and review** | | | | | | |  |
| **F28. Monitor carers’ stress level and provide supportive services** | | | | | | |  |
| Nursing case managers (n=45) | 4 (8.9) |  | 3 (6.7) | 14 (31.1) | 24 (53.3) | 38 (84.4) |  |
| Social worker case managers (n=36) |  |  | 9 (25.0) | 14 (38.9) | 13 (36.1) | 27 (75.0) |  |
| Allied health case managers (n=44) | 4 (9.1) | 1 (2.3) | 7 (15.9) | 20 (45.5) | 12 (27.3) | 32 (72.8) |  |
| Case managers with other professional backgrounds (n=22) | 3 (13.6) |  | 3 (13.6) | 9 (40.9) | 7 (31.8) | 16 (72.7) |  |
| **Total (N=147)** | **11 (7.5)** | **1 (0.7)** | **22 (15.0)** | **57 (38.8)** | **56 (38.1)** | **113 (76.9)** |  |
| **F29. Monitor clients' progress in terms of achieving expected outcomes at specific time frames as defined by care plans** | | | | | | |  |
| Nursing case managers (n=45) |  |  | 3 (6.7) | 21 (46.7) | 21 (46.7) | 42 (93.4) |  |
| Social worker case managers (n=36) | 2 (5.6) | 1 (2.8) | 7 (19.4) | 14 (38.9) | 12 (33.3) | 26 (72.2) |  |
| Allied health case managers (n=44) |  |  | 5 (11.4) | 28 (63.6) | 11 (25.0) | 39 (88.6) |  |
| Case managers with other professional backgrounds (n=22) |  |  | 2 (9.1) | 13 (59.1) | 7 (31.8) | 20 (90.9) |  |
| **Total (N=147)** | **2 (1.4)** | **1 (0.7)** | **17 (11.6)** | **76 (51.7)** | **51 (34.7)** | **127 (86.4)** |  |
| **F30. Maintain ongoing communication with key stakeholders (e.g. care professionals, care providers, clients and carers, etc.) to discuss care plans, service gap, and related issues** | | | | | | |  |
| Nursing case managers (n=45) |  | 1 (2.2) | 2 (4.4) | 15 (33.3) | 27 (60.0) | 42 (93.3) |  |
| Social worker case managers (n=36) | 1 (2.8) |  | 4 (11.1) | 10 (27.8) | 21 (58.3) | 31 (86.1) |  |
| Allied health case managers (n=45) |  |  | 5 (11.1) | 21 (46.7) | 19 (42.2) | 40 (88.9) |  |
| Case managers with other professional backgrounds (n=22) |  |  | 2 (9.1) | 11 (50.0) | 9 (40.9) | 20 (90.9) |  |
| **Total (N=148)** | **1 (0.7)** | **1 (0.7)** | **13 (8.8)** | **57 (38.5)** | **76 (51.4)** | **133 (89.9)** |  |
| **F31. Review clients’ needs and other conditions, as well as their care plans periodically (or as needed), and adjust care plans if necessary** | | | | | | |  |
| Nursing case managers (n=45) | 1 (2.2) |  | 4 (8.9) | 13 (28.9) | 27 (60.0) | 40 (88.9) |  |
| Social worker case managers (n=36) | 1 (2.8) |  | 3 (8.3) | 15 (41.7) | 17 (47.2) | 32 (88.9) |  |
| Allied health case managers (n=45) | 1 (2.2) | 1 (2.2) | 1 (2.2) | 21 (46.7) | 21 (46.7) | 42 (93.4) |  |
| Case managers with other professional backgrounds (n=22) | 1 (4.5) |  | 1 (4.5) | 12 (54.5) | 8 (36.4) | 20 (90.9) |  |
| **Total (N=148)** | **4 (2.7)** | **1 (0.7)** | **9 (6.1)** | **61 (41.2)** | **73 (49.3)** | **134 (90.5)** |  |
| **F32. Monitor care quality and ensure that services are provided appropriately and timely** | | | | | | |  |
| Nursing case managers (n=45) |  |  |  | 14 (31.1) | 31 (68.9) | 45 (100.0) |  |
| Social worker case managers (n=36) | 2 (5.6) |  | 3 (8.3) | 8 (22.2) | 23 (63.9) | 31 (86.1) |  |
| Allied health case managers (n=44) |  |  | 6 (13.6) | 19 (43.2) | 19 (43.2) | 38 (86.4) |  |
| Case managers with other professional backgrounds (n=22) |  |  | 1 (4.5) | 8 (36.4) | 13 (59.1) | 21 (95.5) |  |
| **Total (N=147)** | **2 (1.4)** |  | **10 (6.8)** | **49 (33.3)** | **86 (58.5)** | **135 (91.8)** |  |
| **Outcome evaluation** | | | | | | |  |
| **F33. Develop implementation plans for evaluating the effects of case management plans systematically and periodically** | | | | | | |  |
| Nursing case managers (n=45) | 6 (13.3) | 5 (11.1) | 8 (17.8) | 16 (35.6) | 10 (22.2) | 26 (57.8) |  |
| Social worker case managers (n=36) | 7 (19.4) | 7 (19.4) | 10 (27.8) | 9 (25.0) | 3 (8.3) | 12 (33.3) |  |
| Allied health case managers (n=44) | 9 (20.5) | 10 (22.7) | 13 (29.5) | 10 (22.7) | 2 (4.5) | 12 (27.2) |  |
| Case managers with other professional backgrounds (n=22) | 2 (9.1) | 4 (18.2) | 5 (22.7) | 8 (36.4) | 3 (13.6) | 11 (50.0) |  |
| **Total (N=147)** | **24 (16.3)** | **26 (17.7)** | **36 (24.5)** | **43 (29.3)** | **18 (12.2)** | **61 (41.5)** |  |
| **F34. Evaluate multicultural issues and other factors that influence achieving identified goals and expected outcomes** | | | | | | |  |
| Nursing case managers (n=45) | 1 (2.2) | 7 (15.6) | 13 (28.9) | 13 (28.9) | 11 (24.4) | 24 (53.3) |  |
| Social worker case managers (n=36) | 2 (5.6) | 1 (2.8) | 10 (27.8) | 16 (44.4) | 7 (19.4) | 23 (63.8) |  |
| Allied health case managers (n=44) | 2 (4.5) | 6 (13.6) | 9 (20.5) | 17 (38.6) | 10 (22.7) | 27 (61.3) |  |
| Case managers with other professional backgrounds (n=22) | 1 (4.5) | 1 (4.5) | 6 (27.3) | 10 (45.5) | 4 (18.2) | 14 (63.7) |  |
| **Total (N=147)** | **6 (4.1)** | **15 (10.2)** | **38 (25.9)** | **56 (38.1)** | **32(21.8)** | **88 (59.9)** |  |
| **F35. Evaluate the effects related to identified goals and expected outcomes (e.g. client outcomes, carer outcomes, cost-effectiveness, cost-benefits, etc.) at specified timeframes as defined by care plans** | | | | | | |  |
| Nursing case managers (n=45) | 1 (2.2) | 2 (4.4) | 8 (17.8) | 16 (35.6) | 18 (40.0) | 34 (75.6) |  |
| Social worker case managers (n=36) | 3 (8.3) | 4 (11.1) | 8 (22.2) | 12 (33.3) | 9 (25.0) | 21 (58.3) |  |
| Allied health case managers (n=44) | 3 (6.8) | 3 (6.8) | 10 (22.7) | 20 (45.5) | 8 (18.2) | 28 (63.7) |  |
| Case managers with other professional backgrounds (n=22) |  | 1 (4.5) | 5 (22.7) | 13 (59.1) | 3 (13.6) | 16 (72.7) |  |
| **Total (N=147)** | **7 (4.8)** | **10 (6.8)** | **31 (21.1)** | **61 (41.5)** | **38 (25.9)** | **99 (67.4)** |  |
| **F36. Evaluate the feasibility, timeliness, availability, quality and appropriateness of services identified in care plans** | | | | | | |  |
| Nursing case managers (n=45) | 2 (4.4) | 3 (6.7) | 5 (11.1) | 21 (46.7) | 14 (31.1) | 35 (77.8) |  |
| Social worker case managers (n=36) | 3 (8.3) | 5 (13.9) | 5 (13.9) | 9 (25.0) | 14 (38.9) | 23 (63.9) |  |
| Allied health case managers (n=44) | 3 (6.8) | 3 (6.8) | 11 (25.0) | 15 (34.1) | 12 (27.3) | 27 (61.4) |  |
| Case managers with other professional backgrounds (n=22) |  | 3 (13.6) | 3 (13.6) | 11 (50.0) | 5 (22.7) | 16 (72.7) |  |
| **Total (N=147)** | **8 (5.4)** | **14 (9.5)** | **24 (16.3)** | **56 (38.1)** | **45 (30.6)** | **101 (68.7)** |  |
| **General functions-related activities** | | | | | | |  |
| **F37. Generate client summary reports and present them to key stakeholders (e.g. care professionals, care providers, payers, etc.)** | | | | | | |  |
| Nursing case managers (n=44) | 4 (9.1) | 7 (15.9) | 9 (20.5) | 17 (38.6) | 7 (15.9) | 24 (54.5) |  |
| Social worker case managers (n=36) | 1 (2.8) | 9 (25.0) | 7 (19.4) | 15 (41.7) | 4 (11.1) | 19 (52.8) |  |
| Allied health case managers (n=45) | 5 (11.1) | 5 (11.1) | 15 (33.3) | 9 (20.0) | 11 (24.4) | 20 (44.4) |  |
| Case managers with other professional backgrounds (n=23) | 2 (8.7) | 3 (13.0) | 6 (26.1) | 8 (34.8) | 4 (17.4) | 12 (52.2) |  |
| **Total (N=148)** | **12 (8.1)** | **24 (16.2)** | **37 (25.0)** | **49 (33.1)** | **26(17.6)** | **75 (50.7)** |  |
| **F38. Document the processes of all the core functions as described above and communicate related information to key stakeholders (e.g. clients and carers, care professionals, care providers, payers, etc.)** | | | | | | |  |
| Nursing case managers (n=44) | 4 (9.1) | 1 (2.3) | 4 (9.1) | 14 (31.8) | 21 (47.7) | 35 (79.5) |  |
| Social worker case managers (n=36) | 1 (2.8) |  | 3 (8.3) | 11 (30.6) | 21 (58.3) | 32 (88.9) |  |
| Allied health case managers (n=45) | 1 (2.2) |  | 2 (4.4) | 6 (13.3) | 36 (80.0) | 42 (93.3) |  |
| Case managers with other professional backgrounds (n=23) |  |  | 1 (4.3) | 8 (34.8) | 14 (60.9) | 22 (95.7) |  |
| **Total (N=148)** | **6 (4.1)** | **1 (0.7)** | **10 (6.8)** | **39 (26.4)** | **92 (62.2)** | **131 (88.6)** |  |
| **F39. Ensure that all services are provided within budgetary constraints** | | | | | | |  |
| Nursing case managers (n=44) |  | 1 (2.3) |  | 19 (43.2) | 24 (54.5) | 43 (97.7) |  |
| Social worker case managers (n=36) | 1 (2.8) |  | 2 (5.6) | 17 (47.2) | 16 (44.4) | 33 (91.6) |  |
| Allied health case managers (n=45) |  | 1 (2.2) | 3 (6.7) | 16 (35.6) | 25 (55.6) | 41 (91.2) |  |
| Case managers with other professional backgrounds (n=23) | 1 (4.3) | 1 (4.3) | 1 (4.3) | 6 (26.1) | 14 (60.9) | 20 (87.0) |  |
| **Total (N=148)** | **2 (1.4)** | **3 (2.0)** | **6 (4.1)** | **58 (39.2)** | **79 (53.4)** | **137 (92.6)** |  |
| **F40. Identify and adhere to related guidelines, regulatory requirements, and legislative standards** | | | | | | |  |
| Nursing case managers (n=44) |  |  |  | 7 (15.9) | 37 (84.1) | 44 (100.0) |  |
| Social worker case managers (n=36) |  |  |  | 13 (36.1) | 23 (63.9) | 36 (100.0) |  |
| Allied health case managers (n=45) | 1 (2.2) |  | 1 (2.2) | 9 (20.0) | 34 (75.6) | 43 (95.6) |  |
| Case managers with other professional backgrounds (n=23) | 1 (4.3) |  | 1 (4.3) | 7 (30.4) | 14 (60.9) | 21 (91.3) |  |
| **Total (N=148)** | **2 (1.4)** |  | **2 (1.4)** | **36 (24.3)** | **108 (73.0)** | **144 (97.3)** |  |
| **F41. Maintain clients' privacy, confidentiality and safety** | | | | | | |  |
| Nursing case managers (n=44) |  |  |  | 4 (9.1) | 40 (90.9) | 44 (100.0) |  |
| Social worker case managers (n=36) |  |  | 2 (5.6) | 5 (13.9) | 29 (80.6) | 34 (94.5) |  |
| Allied health case managers (n=45) | 1 (2.2) |  |  | 2 (4.4) | 42 (93.3) | 44 (97.7) |  |
| Case managers with other professional backgrounds (n=23) |  |  |  | 7 (30.4) | 16 (69.6) | 23 (100.0) |  |
| **Total (N=148)** | **1 (0.7)** |  | **2 (1.4)** | **18 (12.2)** | **127 (85.8)** | **145 (98.0)** |  |

Note: The sample size was not 154 due to missing data.
